# Extraperitoneal repair using modified single-port mini-nephroscopy for pediatric hydrocele: surgical outcomes in 65 children

**DOI:** 10.3389/fped.2026.1852266

**Published:** 2026-08-07

**Authors:** Qianliang Wang, Qingling Liu

**Affiliations:** 1 Department of Urology, Affiliated Hospital of Guangdong Medical University, Zhanjiang, China; 2 Department of Medical Records Room, Affiliated Hospital of Guangdong Medical University, Zhanjiang, China

**Keywords:** high ligation of the patent processus vaginalis, hydrocele, minimally invasive surgical, mini-nephroscopy, ultrasound

## Abstract

**Objective:**

The purpose of the study is to introduce our experience of a modified single-port Mini-Nephroscopy technique for the treatment of pediatric hydrocele.

**Methods:**

In this prospective study, 65 male children (age range: 1.5–8 years) with unilateral communicating hydrocele were enrolled between June 2019 and June 2022. The surgical procedure consisted of high ligation of the patent processus vaginalis performed extraperitoneally using a modified single-port nephroscope introduced through a single umbilical incision, facilitated by custom suture-guiding instruments. The primary outcome measures included testicular volume and testicular artery resistance index (RI), which were evaluated via scrotal ultrasonography preoperatively and at scheduled follow-ups of 1 and 6 months.

**Results:**

The novel technique was successfully employed in all 65 patients without conversion to open surgery. The mean operative time was remarkably brief (8–10 min), facilitating a 24-hour discharge for all children. Postoperative pain was well-controlled, with mean VAS scores decreasing from 2.1 ± 0.82.1 ± 0.8 at 6 h to 1.2 ± 0.51.2 ± 0.5 at 24 h, indicating only mild and transient discomfort. With a follow-up of 3–18 months, no instances of recurrence, testicular atrophy, or other complications (e.g., knot reactions, iatrogenic ascent) were observed.

**Conclusion:**

Single-port nephroscopic high ligation holds significant promise for pediatric communicating hydrocele, offering a compelling combination of minimal invasiveness, high operative efficiency, rapid recovery, and an excellent safety profile. These results strongly endorse its consideration as a first-line surgical treatment for this condition.

## 1 Introduction

Communicating hydrocele represents one of the most prevalent pediatric urological conditions, pathophysiologically rooted in the persistence of a patent processus vaginalis (PPV) due to developmental failure of closure. This anatomical communication permits the continuous passage of peritoneal fluid into the scrotal cavity, disrupting testicular hemodynamics and microcirculation. If left untreated, this condition may progress to complications such as testicular atrophy, underscoring the importance of timely intervention (1). While spontaneous resolution occurs in a proportion of infants under 12 months of age, surgical correction becomes the standard of care beyond this age to avert long-term morbidity.

The current surgical management of communicating hydroceles primarily encompasses two approaches: the traditional open high ligation via an inguinal incision and the laparoscopic transabdominal closure of the PPV. Both techniques share the common objective of complete ligation of the processus vaginalis to eliminate the pathological communication between the peritoneal cavity and the tunica vaginalis. Although laparoscopic surgery is recognized as less invasive than open surgery, conventional laparoscopic approaches typically necessitate 2–3 trocar incisions. This multi-port requirement not only increases procedural complexity but also elevates the potential for port-related complications and cosmetic concerns.

In response to these limitations, our institution pioneered the introduction of single-port nephroscopic high ligation (SPN-HL) for pediatric communicating hydrocele in June 2019. This innovative technique represents a significant evolution in minimally invasive pediatric urologic surgery. The SPN-HL approach offers several paradigm-shifting advantages: (1) it is ultra-minimally invasive, requiring only a single 5-mm umbilical incision; (2) it utilizes simplified instrumentation through a specialized pediatric nephroscope with integrated suture guidance capability; and (3) it provides enhanced surgical precision through direct magnified visualization and controlled ligation of the PPV in the extraperitoneal space. This comprehensive report elaborates our institutional experience with SPN-HL, detailing the technical methodology, perioperative outcomes, and follow-up results, thereby demonstrating its clinical viability and potential superiority over conventional surgical approaches.

### 1.1 Patient selection and demographic characteristics

From June 2019 to June 2022, a prospective cohort of 65 male pediatric patients diagnosed with unilateral communicating hydrocele was enrolled in this study, which was registered with the Chinese Clinical Trial Registry (Registration number: ChiCTR1900055564). The mean age of the cohort was 32.6 months, with a range from 18 to 96 months. Diagnosis was established through a standardized protocol involving clinical evaluation by two independent pediatric urology specialists, complemented by confirmatory color Doppler ultrasonography. The cohort comprised 20 left-sided and 45 right-sided cases (Table 1). All participants were otherwise healthy, with no documented comorbidities or previous surgical history involving hernia repair or other inguinoscrotal procedures.

**Table 1:** Preoperative and postoperative general data of patients.

Variables	Values
Cases (NO.)	65
average age (months)	32.6 (18–96)
Left side (NO.)	20
Right side (NO.)	45
Mean operative time (min)	9.2 (8–10)
Intraoperative bleeding (mL)	0
average stay (h)	24

Comprehensive preoperative assessment included bilateral scrotal ultrasonography performed by experienced sonographers using a 14 MHz linear array transducer. The examination protocol incorporated precise measurement of testicular arterial blood flow parameters, including peak systolic velocity (PSV) and end-diastolic velocity (EDV). The resistance index (RI) was subsequently calculated according to the standard formula: RI = (PSV − EDV)/PSV × 100. Additionally, meticulous volumetric assessment of both testes was conducted to establish baseline morphological parameters (2, 3).

The study adhered to stringent inclusion and exclusion criteria to ensure cohort homogeneity. Inclusion criteria encompassed: (1) age between 1.5 and 8 years; (2) definitive diagnosis of communicating hydrocele of the spermatic cord confirmed by both clinical and ultrasonographic evaluation; (3) presence of clear surgical indications based on established clinical guidelines; and (4) provision of written informed consent by legal guardians. Exclusion criteria comprised: (1) secondary hydrocele resulting from underlying pathologies including neoplasms, ascites, trauma, infection, or torsion of testicular appendages; (2) concomitant conditions necessitating additional surgical interventions such as inguinal hernia or cryptorchidism; (3) contraindications to general anesthesia or surgical intervention; and (4) history of previous ipsilateral inguinal or scrotal operations.

### 1.2 Procedure of single-port mini-nephroscope surgical technique

Following bladder emptying, general anesthesia with endotracheal intubation was administered. The patient was positioned in the Trendelenburg position. A 5-mm skin incision was made at the medial aspect of the inferior umbilical border. The abdominal wall was elevated, and a Veress needle was introduced into the peritoneal cavity to establish pneumoperitoneum. After needle removal, a single 4-mm port was placed. Pneumoperitoneum pressure was maintained between 8 and 10 mmHg. A nephroscope was inserted to inspect for potential visceral injury and to assess the patency of the processus vaginalis bilaterally before proceeding with the surgical intervention.

Using the right side as an exemplar for the procedure: A 1-mm skin puncture was created slightly lateral to the surface projection of the internal ring, serving as the external entry point. To minimize the risk of injuring the spermatic vessels and vas deferens, a saline-filled syringe was used to penetrate the abdominal wall from this external point into the peritoneal cavity. Under nephroscopic visualization, careful dissection was performed to identify the retroperitoneal space, spermatic vessels, and vas deferens.

A hernia needle carrying a 3-0 non-absorbable suture was introduced at the 12 o'clock position of the external puncture site, entering the medial aspect of the patent processus vaginalis within the extraperitoneal space. Under direct nephroscopic guidance, the needle was advanced superior to the spermatic vessels and vas deferens. In cases of prominent tissue folds, the auxiliary forceps of the nephroscope were utilized to facilitate needle passage. Upon reaching the approximately 6 o'clock position, the needle was advanced through the peritoneum into the abdominal cavity. The needle was then withdrawn while grasping the suture end, leaving it within the abdomen and the suture tail external to the abdominal wall, thereby completing the medial semicircular suture around the processus vaginalis. The needle was withdrawn and reinserted through the original cutaneous puncture to similarly complete the lateral semicircular suture.

**Figure 1:**
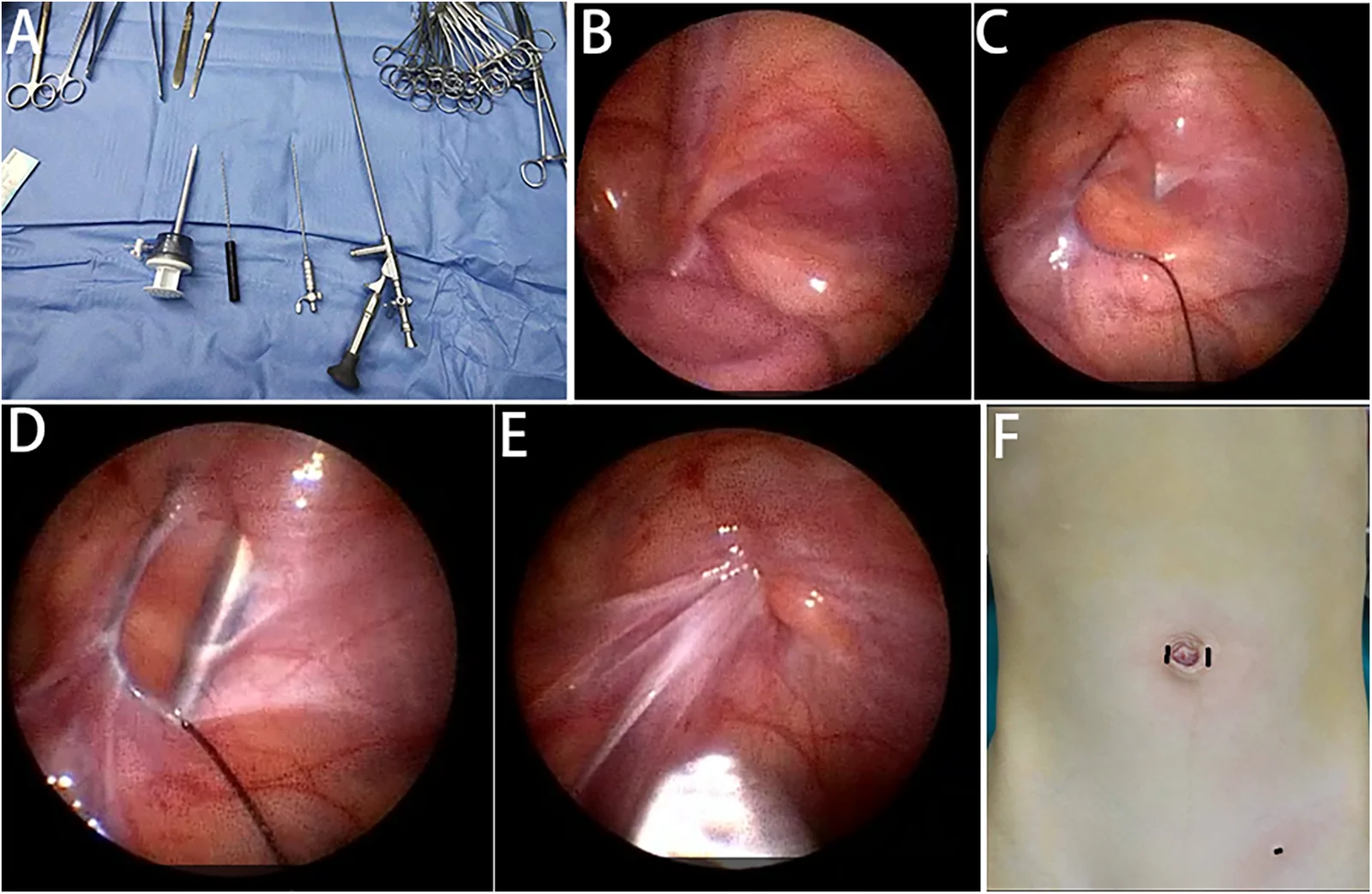
Surgical instruments and intraoperative and postoperative outcomes. **(A)** The surgical instruments required during the procedure include the STROZ nephroscope and the hernia needle. **(B)** Dysfunction or incomplete closure of the processus vaginalis. **(C)** The medial needle entry completes the medial half-circular suture. **(D)** The lateral needle entry completes the medial half-circular suture. **(E)** After the circular purse-string suture, the closure of the internal ring is evident. **(F)** The postoperative incision site is shown.

The accumulated hydrocele fluid was either manually reduced into the peritoneal cavity or aspirated via scrotal puncture. With the assistance of the auxiliary forceps, the suture was externalized. The two external suture ends were elevated and tied, accomplishing a circumferential high ligation of the patent processus vaginalis. The knot was buried subcutaneously, and the external puncture site required no skin suture. The umbilical incision was sealed with surgical adhesive, concluding the procedure (Figure 1 for surgical details).

No postoperative intravenous antibiotics or analgesics were administered. Patients were scheduled for discharge 24 h after surgery, following confirmation of stable vital signs and successful oral intake.

To systematically evaluate postoperative recovery and reproductive organ health, all patients underwent standardized scrotal ultrasound examinations at 1- and 6-month intervals. These assessments specifically measured testicular volume and testicular artery resistance index (RI) to objectively monitor testicular perfusion and development.

A comprehensive follow-up protocol was implemented for all patients, extending to 18 months postoperatively. This extended surveillance period allowed for thorough documentation of functional outcomes, detection of potential late complications, and assessment of long-term surgical efficacy.

### 1.3 Observation indicators and efficacy evaluation

#### 1.3.1 Perioperative efficiency and safety parameters

Operative Time: The total duration from initial skin incision to final skin closure was recorded (minutes). Intraoperative Blood Loss: Volume was recorded via visual estimation of blood on gauze and in suction apparatus (mL). Given the typically minimal blood loss in pediatric surgeries, this was qualitatively categorized as “minimal, <5 mL” or “negligible”. Intraoperative Complications: Any adverse intraoperative events were documented, including but not limited to injury to the vas deferens, spermatic cord vessels, peritoneal breach, or intestinal injury.

Postoperative Hospital Stay: The total duration from the conclusion of surgery until the patient met predefined discharge criteria (characterized by stable vital signs, tolerance of oral diet, a dry incision without exudate, and unrestricted ambulation) was recorded (hours or days).

#### 1.3.2 Technical success and short-term efficacy metrics

Procedural Success Rate: Defined as the successful laparoscopic identification and high ligation of the patent processus vaginalis without conversion to open surgery.

Incision Length and Cosmesis: The postoperative incision length was measured precisely (mm). At the 1-month follow-up, cosmetic outcome was assessed by parents using either a Visual Analog Scale (VAS) or the Vancouver Scar Scale (VSS).

Early Postoperative Complications (≤30 days): All complications occurring within 30 days postoperatively were documented and graded in detail, encompassing:

Incision-related: Infection, hematoma, impaired healing.

Scrotal-related: Scrotal edema, hematoma.

Systemic/Other: Fever, urinary retention, among others.

#### 1.3.3 Primary efficacy endpoint: recurrence rate

Assessment Method: Evaluation was performed via clinical physical examination and color Doppler ultrasonography.

Assessment Time Points: Scheduled follow-up assessments were conducted at 3, 6, and 18 months postoperatively.

Definition of Recurrence: The reappearance of a sonographically or clinically confirmed fluid collection in the ipsilateral scrotum during the follow-up period, which demonstrated no significant reduction with postural change or over time.

#### 1.3.4 Safety evaluation: testicular function and development

Testicular Volume and Vascularization: Testicular volume (affected and contralateral sides) and testicular artery blood flow parameters (Resistive Index, Pulsatility Index) were evaluated using color Doppler ultrasonography preoperatively and at 6 months and/or 1 year postoperatively.

Testicular Position: Postoperative testicular retraction or ascent was monitored.

#### 1.3.5 Postoperative recovery and patient experience measures

Postoperative Pain Scores: Assessed at 6 and 24 h postoperatively using the FLACC (Face, Legs, Activity, Cry, Consolability) scale (for preverbal children) or the Wong-Baker FACES Pain Rating Scale.

Postoperative Analgesic Use: The requirement for, specific agent used (e.g., acetaminophen, ibuprofen), and frequency of administration of analgesic medications within the first 24 h postoperatively were recorded.

Time to Resume Normal Activities: The number of days required for the child to return to normal daily play and learning activities was documented.

### 1.4 Statistical analysis

Quantitative data were subjected to analysis using SPSS software (Version 19.0, Chicago, IL, USA) and were presented as means ± standard error of the mean (SEM). Statistical comparisons between groups were conducted using ANOVA, followed by a Student-Newman-Keuls (SNK-q) test for *post-hoc* analysis. A *p*-value less than 0.05 was considered to be statistically significant.

## 2 Results

All surgical procedures were successfully completed using the modified single-port nephroscopic technique without conversion to open surgery. No intraoperative complications occurred, including injury to spermatic vessels, vas deferens, gonadal blood supply, or abdominal organs.

Postoperative outcomes demonstrated a favorable safety profile. Three patients (4.6%) developed transient scrotal emphysema that resolved spontaneously within 48 h, while two patients (3.1%) experienced mild scrotal edema that subsided without intervention within 2 weeks. No cases of wound infection, testicular hemorrhage, or other early complications were observed. Postoperative pain was effectively managed, with FLACC scale scores decreasing from 2.1 ± 0.8 at 6 h to 1.2 ± 0.5 at 24 h postoperatively (Table 2). Throughout the follow-up period ranging from 3 to 18 months, no instances of incisional hernia, testicular atrophy, or hydrocele recurrence were documented.

**Table 2:** Postoperative complications: type and incidence.

Parameter	*N* (%)
Scrotal emphysema (NO.)	3 (4.6%)
Mild scrotal edema (NO.)	2 (3.1%)
Vascular injury (NO.)	0
Orchitis/epididymitis (NO.)	0
Wound infection (NO.)	0
Postoperative pain (FLACC score)	2.1 ± 0.8 (at 6 h)
	1.2 ± 0.5 (at 24 h)
Late complications, *n*	0
Recurrence	0
Testicular atrophy	0

During the initial diagnostic phase, contralateral patent processus vaginalis (PPV) was identified in 7 patients (10.8%), resulting in a total of 72 high ligation procedures performed among the 65 patients. Postoperative scrotal ultrasonography at 1 and 6 months confirmed the absence of vas deferens torsion or constriction in all cases.

Quantitative analysis of testicular parameters revealed preserved testicular function and development following surgical intervention. In the 58 patients who underwent unilateral high ligation, no statistically significant differences were observed in testicular volume or resistance index (RI) between preoperative values and measurements obtained at 1- and 6-month follow-up intervals, either on the operated or contralateral side (Table 3). Similarly, among the 7 patients who underwent bilateral procedures, comparative analysis demonstrated no significant alterations in testicular volume or vascular parameters between the diagnostic and PPV sides at both follow-up timepoints (Table 4).

**Table 3:** Testicular resistance index (RI) and volume of the pediatric patients before and after unilateral inguinal hernia repair (*n* = 58).

	Testicular RI			Testicular volume (mL)		
	Preop.	1 month postop.	6 months postop	Preop	1 month postop	6 months postop
Surgical side	0.59 ± 0.06	0.58 ± 0.07	0.58 ± 0.06	1.02 ± 0.14	0.99 ± 0.17	0.98 ± 0.16
Contralateral side	0.58 ± 0.07	0.57 ± 0.06	0.57 ± 0.59	0.98 ± 0.16	1.01 ± 0.17	1.03 ± 0.14
*p*	0.201	0.495	0.784	0.135	0.086	0.315

**Table 4:** Testicular resistance index (RI) and volume of the pediatric patients before and after bilateral inguinal hernia repair (*n* = 7).

	Testicular RI		Testicular volume (mL)	
	Diagnosed side	PPV side	Diagnosed side	PPV side
Pre-operatively	0.58 ± 0.06	0.59 ± 0.04	0.99 ± 0.15	0.98 ± 0.08
1 months postoperatively	0.58 ± 0.04	0.58 ± 0.07	0.98 ± 0.10	0.99 ± 0.05
6 months postoperatively	0.58 ± 0.06	0.57 ± 0.05	0.96 ± 0.12	0.99 ± 0.08
*p*	0.754	0.215	0.703	0.584

## 3 Discussion

The pathogenesis of pediatric communicating hydrocele originates from embryological development anomalies, specifically the persistent patency of the processus vaginalis (PV), which establishes an abnormal communication channel between the peritoneal cavity and the scrotum (4). During normal male fetal development, the testes descend from the abdominal cavity through the inguinal canal around the seventh month of gestation, trailed by the PV. Under physiological conditions, this tubular structure undergoes spontaneous fibrous obliteration during the first year of life. However, when this closure mechanism fails, it creates a pathway for peritoneal fluid accumulation within the scrotal cavity under gravitational forces (5). The clinical significance of this condition extends far beyond simple fluid collection. Elevated intracystic pressure can compromise testicular microcirculation through a compartment-like effect, while persistent fluid accumulation may alter the local thermal environment, potentially affecting spermatogenic function and testicular development (6, 7). Contemporary research indicates that prolonged hydrocele presence can lead to measurable changes in testicular parenchyma, emphasizing the importance of timely intervention (8).

The natural history of communicating hydrocele demonstrates that spontaneous resolution occurs in approximately 80% of infants within the first 12 months. However, beyond 18 months, the likelihood of spontaneous closure decreases dramatically to less than 5%, making surgical intervention the standard of care (9). The timing of surgery represents a critical clinical decision, balancing the potential for spontaneous resolution against the risk of long-term testicular dysfunction. Current guidelines recommend surgical intervention for persistent hydroceles beyond 18–24 months, though this may be individualized based on hydrocele size and progression (10).

The surgical management paradigm for communicating hydrocele has undergone substantial evolution, transitioning from traditional open approaches to increasingly sophisticated minimally invasive techniques. Conventional open repair, while effective in experienced hands, necessitates inguinal dissection through a 2–3 cm incision, with associated postoperative discomfort, longer recovery periods, and visible scarring (11). The introduction of laparoscopic techniques in the 1990s marked a revolutionary advancement, providing magnified visualization of the internal ring and surrounding critical structures, including the vas deferens and spermatic vessels (12, 13). This improved visualization theoretically reduces the risk of iatrogenic injury to these delicate structures, though it introduces new technical challenges and equipment requirements.

The chronological progression of laparoscopic PV ligation has encompassed three distinct technological generations, each representing significant technical refinements. The initial three-port techniques, pioneered in the early 2000s, utilized separate working channels for needle holders and operative forceps, facilitating circumferential suturing and ligation of the PV (14). While effective, this approach required multiple abdominal incisions, increasing the complexity of the procedure and potential for port-related complications. The subsequent development of two-port approaches represented a meaningful advancement, reducing the number of required incisions while maintaining procedural efficacy (15). However, this technique still necessitated an auxiliary instrument through a separate incision, limiting its minimally invasive potential. The most recent evolution to single-port methodologies represents the current frontier in minimally invasive pediatric urologic surgery, though early implementations faced significant technical hurdles (16).

Traditional three-port laparoscopic surgery, while representing a substantial improvement over open techniques, carries inherent limitations related to multiple abdominal incisions. Contemporary meta-analyses indicate that multiport laparoscopy is associated with a 3.2%–4.1% risk of port-site complications, including hemorrhage, infection, and late-onset hernia formation (17). Additionally, the cosmetic outcomes, while superior to open surgery, still involve multiple visible scars. The two-port method, while reducing the number of incisions, still requires careful triangulation of instruments and may present challenges in certain anatomical configurations. Early single-port attempts addressed the cosmetic concerns but introduced new technical difficulties, particularly regarding instrument triangulation, suture manipulation, and the frequent collisions between instruments, often resulting in prolonged operative times and steep learning curves (18).

Our modified single-port nephroscopic technique represents a significant evolution beyond conventional laparoscopic approaches, specifically addressing the limitations of previous single-port methodologies. By utilizing the specialized STORZ nephroscope (2.7–3.8 mm diameter) with its integrated dual-channel design, this approach achieves complete extraperitoneal PV ligation through a single umbilical incision. The instrument's optimized dimensions and 30-cm working length are particularly suited to pediatric anatomy, effectively addressing the limitations of conventional laparoscopes that may cause disproportionate tissue trauma in children. The nephroscope's unique configuration allows for both visualization and instrument manipulation through a single access point, eliminating the instrument crowding that plagued early single-port attempts.

Comparative analysis reveals several distinct advantages of our technique over established multiport approaches. The reduction to a single 4-mm access point results in substantially less abdominal wall trauma, translating into significantly improved postoperative pain profiles. Our data demonstrate mean VAS scores of 2.1 ± 0.8 at 6 h, decreasing to 1.2 ± 0.5 at 24 h, comparing favorably with conventional multiport techniques (reported VAS 3.5 ± 1.2 at 6 h, 2.8 ± 1.1 at 24 h) (19). Procedural efficiency represents another significant advantage, with operative times of 8–10 min comparing favorably with reported durations for three-port (15–25 min) and two-port (12–18 min) techniques (20). This improved efficiency likely reflects the streamlined instrumentation and reduced time required for multiple port placements.

The integrated suture guidance system enables precise extraperitoneal dissection and circumferential suturing under magnified visualization, significantly minimizing risk to the vas deferens and spermatic vessels. This addresses a significant concern in multiport techniques where instrument crowding and limited working space may impair visualization and increase the risk of iatrogenic injury (21). Our extraperitoneal approach represents another key advantage, minimizing peritoneal irritation and potentially reducing postoperative discomfort compared to transperitoneal techniques. This approach also eliminates the risk of intra-abdominal organ injury and reduces the likelihood of postoperative adhesive disease.

Our outcomes demonstrate excellent safety and efficacy profiles that compare favorably with conventional approaches. The complete absence of recurrences or testicular atrophy during the 3–18 month follow-up period aligns with or surpasses outcomes reported for established techniques. Particularly noteworthy is the complete absence of iatrogenic testicular ascent or vasal injury, complications documented in 1.2%–2.1% of cases in larger laparoscopic series. The observed minor complications (transient scrotal emphysema: 4.6%; mild scrotal edema: 3.1%) resolved spontaneously without intervention, representing a favorable profile compared to port-related complications in multiport approaches (22). These findings suggest that our technique maintains the efficacy of conventional approaches while potentially offering improved safety profiles.

The technical success of our method specifically addresses several limitations of previous single-port attempts. The nephroscope's integrated operating channel facilitates precise needle manipulation without the spatial constraints and instrument collisions that complicated conventional single-port laparoscopy. The ability to maintain stable pneumoperitoneum through a single port represents another technical refinement, addressing a common challenge in single-port surgery. Furthermore, the extraperitoneal approach not only minimizes peritoneal irritation but also provides enhanced visualization of the PV and surrounding structures, potentially contributing to the procedure's efficacy and safety.

While our experience demonstrates promising results, several limitations warrant consideration in interpreting these findings. The single-center design and moderate sample size, while sufficient for initial technical assessment, necessitate validation through larger, multicenter randomized trials. The non-randomized design introduces potential selection bias, though our consecutive enrollment approach mitigates this concern to some extent. Additionally, the technical learning curve, while manageable in our experience, requires systematic training in single-port instrumentation and suturing techniques. Future investigations should focus on prospective randomized comparisons with conventional techniques, long-term testicular function assessment using sensitive parameters including semen analysis in post-pubertal patients, and comprehensive cost-effectiveness analyses to establish broader applicability. The generalizability of our technique may be influenced by equipment availability and surgeon expertise, particularly in resource-limited settings.

## 4 Conclusion

In conclusion, our preliminary experience demonstrates that modified single-port nephroscopic high ligation of the processus vaginalis is a feasible, safe, and highly effective technique for the treatment of pediatric communicating hydrocele. This approach offers distinct advantages, including minimal invasiveness through a single umbilical incision, a remarkably short operative time, rapid postoperative recovery, and excellent cosmetic outcomes. The absence of recurrence or significant complications in our cohort, coupled with preserved testicular volume and vascularization, underscores the procedural safety and efficacy. These findings strongly support the adoption of this technique as a promising surgical alternative for pediatric communicating hydrocele. Further multicenter studies with larger sample sizes and extended follow-up are warranted to validate its long-term benefits and broader applicability.

## Data Availability

The original contributions presented in the study are included in the article/Supplementary Material, further inquiries can be directed to the corresponding author.
